# The Use of an Optical Measurement System to Monitor Sports Performance

**DOI:** 10.3390/sports6010015

**Published:** 2018-02-17

**Authors:** Eric D. Magrum, John P. Wagle, Brad H. DeWeese, Kimitake Sato, Michael H. Stone

**Affiliations:** 1 Department of Kinesiology, University of Georgia, Athens, GA 30602, USA; 2 Department of Sport, Exercise, Recreation, and Kinesiology, East Tennessee State University, Johnson City, TN 37601, USA; Waglej@etsu.edu (J.P.W.); dlive11@gmail.com (B.H.D.); satok1@etsu.edu (K.S.); stonem@etsu.edu (M.H.S.)

**Keywords:** ground contact time, Optojump, force platform, sprint, jumping, athletic performance

## Abstract

The purpose of this study was to compare ground contact time between an optical measurement system and a force platform. Participants in this study included six collegiate level athletes who performed drop jumps and sprint strike steps for a total of 15 repetitions each. Ground contact data was simultaneously collected from an optical measurement system and a force platform, at a sampling frequency of 1000 Hz. Data was then analyzed with Pearson’s correlation and paired sample *t*-tests. The measures from the optical measurement system were found to be significantly higher (*p* < 0.001) than measures from the force platform in both conditions. Although significantly different, the extremely large relationships (0.979, 0.993) found between the two devices suggest the optical sensor is able to detect similar changes in performance to that of a force platform. Practitioners may continue to utilize optical sensors to monitor performance as it may provide a superior user-friendly alternative to more traditional based monitoring procedures, but must comprehend the inherent limitations due to the design of the optical sensors.

## 1. Introduction

With rising interest in sport science, monitoring physical output has become more prevalent. Additionally, the ways in which performance can be directly examined is still being refined for many different sports. Ground contact time has been previously used to monitor performance in activities such as sprinting [1], plyometrics [2], and even endurance events [3] due to its established relationship with performance outcomes. 

The world’s best sprinters have been found to spend shorter time on the ground when compared to sub-elite sprinters [4]. At top speed, 0.087 s is all the time required for a world class sprinter to produce enough force to project their body into the next stride. Furthermore, findings suggest better sprinters produce more force in the first half of their contact time (0.05 s), when compared to their counterparts [5]. Therefore, examining ground contact time over the course of training may provide coaches and researchers with a deeper understanding of the athletes’ physical capacities in sprinting. Furthermore, this information can be used to improve training plans with the goal of enhancing sprint performance, and may also serve to monitor a training program’s effectiveness. 

The current gold standard for measuring ground contact time (GCT) is the use of a force platform [6]. Both force platform and high speed camera have been used to capture GCT while sprinting [5,7,8]. In these studies, a specialized high-speed treadmill equipped with a force platform measured GCT in top speed running. Other investigations [2] utilize force platforms to measure GCT in drop jumps to see the effect on power, work, and moment. With established interest and investigations measuring GCT from either force platforms or high speed video, other devices have been engineered to measure GCT but also generate readily available information for coaches. Progressing performance relies on valid and reliable measures. Therefore, before implementing any measuring device limitations of use must be understood. 

While the use of a force platform or high speed video system is common practice in the current literature, there are limitations to their use. First, neither of the aforementioned options provide coaches time-efficient collection, analyses, and feedback to augment training. Each of the investigations listed above indicate timely equipment setup for collection, analysis requiring specialized software and skillset, and reporting which may pose problems for coaches needing the information quickly. Secondly, investigations above indicate lab-based protocols or indoor facilities requiring large quantities of space. In most situations, coaches are limited on availability, space, and time, making high speed video or force platform utilization inefficient and a potentially denigrating to routine training.

The Optojump (Microgate, Bolzano, Italy) has been used to investigate vertical jump height [9], flight time [9,10], stride length [10], stride frequency [10], sprint velocity [10], ground contact time [10], stride velocity [10], and stride rhythm [10]. These variables are calculated from interferences in optical sensors encased in each transmitting and receiving bar. Findings from these investigations suggest that the Optojump is a valid option for the collection and analysis of jump performance, however, the brief ground contact times required for high-level sprinting have yet to be compared to a criterion measure. 

Optical sensor devices may make monitoring ground contact time more accessible and provide an alternative means by which to monitor other sporting tasks. Optical sensor devices may provide coaches and sport scientists with a more time efficient equivalent to the common lab based testing that is usually predicated on the use of high speed camera or force platform monitoring. In an effort to replicate and further investigate measurement validity, the current study utilized a similar design to Healy, Kenny & Harrison [6]. The aforementioned study aimed to assess the concurrent validity of the Optojump system in estimation of ground contact time and reactive strength measures when compared with a force platform. Results from this investigation indicate an overestimation in contact time and underestimation in flight time in the Optojump. It is important to note that this investigation did not investigate contact times similar to those seen in sprinting. Findings from this investigation are in agreement with and corroborate other investigations [2] conclusions indicating the Optojump is an appropriate measurement device when used for jumping performances.

In an effort to extend the Optojump’s accepted measurement, emphasis in the present study was placed on the brief contacts associated with high-level sprinting and was the first to investigate the sprint strike step. The sprint strike step is a modification from the A-Skip [11], where the athlete balances on one foot while the other foot strikes the ground. Therefore, the primary purpose of this study was to compare the foot contact time between an optical sensor system and a force platform. In accordance with previous work [6], we hypothesize ground contact time will be overestimated by the optical measurement system.

## 2. Materials and Methods

Participants were healthy, young adults who volunteered for the study (*N* = 6). Inclusion criteria was set to include collegiate level athletes who were free of injuries, at least 18 years of age, and able to perform five consecutive jumps. It is important to note that this study was designed to compare the measurements of two devices. Therefore, details of participants’ physical or athletic characteristics were not a part of the study criteria, as those should not affect the comparison process. Study information was provided to all potential, eligible individuals verbally prior to voluntary participation. The study protocol was approved by the University’s Institutional Review Board.

The OptoJump Next system (revision, Microgate, Bolzano, Italy) was the device measured for validity. This measurement system utilizes 32 infrared LED’s sampling at 1000 Hz encased within transducer and receiving bars (1 m each). When an LED light is disturbed, the timer in the unit is triggered and records with a precision of 1 millisecond [6]. Portable force platforms (Pasco, Redding, CA, USA) were used to compare foot contact time against the OptoJump Next system and utilized specialized software to extract data (Pasco Capstone, 1.5), while OptoJump Next software was used in accordance with the optical system. To determine contact time, a level of force <5 N was used as a threshold for take-off and landing was assigned the time point which the force was ≥5 N [12].

It is worth noting that the sensing unit contained within the optical sensor rests just above the running surface (0.003 m) and may pose problems when comparing to force platforms, residing slightly beneath them. Multiple force platforms were used to ensure a sufficient landing area for the participants’ safety. 

Participants were taken through a standardized dynamic warm-up before being able to perform drop jumps and sprint strike step. Two tests were selected for all athletes to perform (1) drop jump and (2) sprint strike step for a total of 15 repetitions (3 sets of 5 repetitions) for each condition. A total of 180 contacts were measured between the two devices. Drop jumps were performed from a 12″ box (30.48 cm) and athletes were instructed to “ricochet” of the ground to minimize ground contact. This movement was used because of participant familiarity and its emphasis to minimize ground contact. The sprint strike step was used to mimic brief ground contact times observed in sprinting, without requiring maximal sprint efforts. Both instruments were simultaneously sampling at 1000 Hz during data collection.

Paired sample *t*-tests were conducted to compare the measurements between two measuring devices for each condition. Cohen’s *d* with 95% confidence intervals (CI) were calculated from mean differences and were used to determine the magnitude of difference. Effect size (ES) values were interpreted as trivial (0.0), small (0.2), moderate (0.6), large (1.2), very large (2.0), and nearly perfect (4.0) [13]. Means and standard deviations were calculated and are located in Table 1 and Table 2. Thresholds for Pearson correlations were based on the following scale: small, 0.10; moderate, 0.3; large, 0.5; very large, 0.7; extremely large, 0.90 [14]. Significance level was set at 0.05. 

## 3. Results

Excluding outliers, a total of 175 foot contacts were used to compare the two devices. Two data points were considered outliers in sprint strike step data. Thus, total 88 data points were considered for the analysis. Three data points were considered outliers in the drop jump condition, a total of 87 data points were considered for analysis. A data point was classified as an outlier if it was found to be greater than two standard deviations outside of the mean difference. Results of analysis and descriptive data are displayed in Table 1 and Table 2. Although there is extremely large correlations for both conditions, paired sample *t*-tests showed the differences between the two devices were statistically significant (sprint strike step, *p* < 0.001, 95% CI (0.0075–0.0096), ES 0.36; drop jump, *p* < 0.001, 95% CI (0.0121–0.0144), ES 0.36). The two measuring devices had a mean difference of 0.009 +/− 0.005 s during the sprint strike step (Table 1), and 0.014 +/− 0.005 s during drop jump (Table 2).

**Figure 1:**
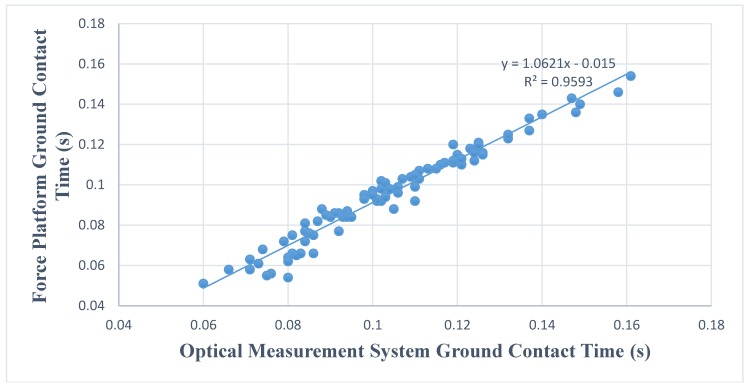
Relationship between ground contact time between force platform and optical sensor during sprint strike step.

**Figure 2:**
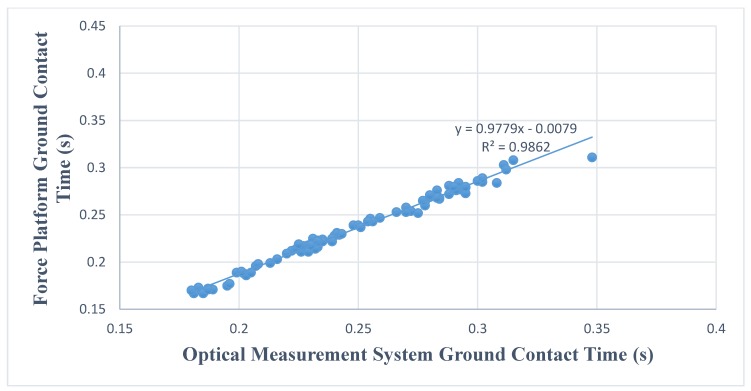
Relationship between ground contact time between force platform and optical sensor during drop jump.

Figure 1 and Figure 2 show the relationship and regression equation on both testing conditions. Very high R^2^ scores are present in this study showing the relationship of the data from both devices (Figure 1 and Figure 2).

## 4. Discussion

The purpose of this study was to compare contact time between an optical measurement system and a force platform. Findings from previous investigations [15] have found contact time of the optical sensor was only moderately valid when compared with a high-speed camera. When compared with a force platform, investigations [6] have found contact time to be overestimated by the optical sensor. Another study compared contact time between two optical systems and found the differences between the two devices were trivial and clinically meaningless [16]. Findings from the current study support previous evidence [6], suggesting that the optical sensor measures statistically higher values of contact time, when compared to a force platform. 

Although found to be significantly different, extreme positive linear relationships existed between the two measuring devices. In addition, R^2^ (0.959, 0.986) values indicate the strength of the relationship between the two devices is nearly perfect [17]. It is important to note magnitudes of difference were small rather than trivial for both conditions (0.36, 0.36). While evidence is not conclusive, the nearly perfect and extremely similar measurements from both devices may be indicative of the optical sensor’s capability of detecting similar changes to that of a force platform. Therefore, the optical sensor may be useful in monitoring performance, despite the statistical difference found in the current investigation. 

Optical sensors have been used to measure jump height [9,12], stiffness [16], and explosive lower body strength [18]. This is the first investigation to compare brief contacts (<0.100 s) between an optical sensor and a force platform. Results may provide the impetus for the inclusion of optical sensors as a monitoring tool for movements with brief contact times. Track and field coaches, as well as other coaches who value sprinting speed and contact time, may use optical sensors to gauge how their strength and on-field training transfers to acceleration or maximal velocity capabilities. 

To monitor an entire team’s performance with embedded force platforms or high-speed video may come at the expense of a full-time sport scientist, a fully equipped laboratory, and time intensive analysis. In contrast, practitioners who adopt optical sensors may gain similar information and save time and expertise.

Findings from this investigation support conclusions from previous work [6], and further suggest the design and elevation of optical sensors above the running or playing surface may be the cause of overestimations in contact time. 

## 5. Conclusions

Evidence supports the original hypothesis, stating that contact time will be overestimated by the optical measurement system when compared to a force platform. Furthermore, the current study suggests that coaches may confidently use the optical measurement systems to monitor performance. Due to the extremely similar relationship found between force platforms and the optical sensor, the current evidence suggests optical sensors have the capability to detect similar changes in performance, when compared to a force platform.

Optical sensors may help coaches monitor performance in the field and make informed decisions, with minimal time taken away from training. The demonstrated ability to detect small changes in performance provides further justification for coaches to utilize these tools. These practices may enhance coaches’ knowledge, alter the training regimen, and improve the overall training experience for athletes. Practitioners who utilize many instruments to monitor performance should be aware of the devices they use, as they may not measure performance in the same manner. When using different devices to measure specific performances, caution should be used in comparing the raw values. Coaches are urged to continue to use optical systems to monitor performance as it provides a potentially superior, user-friendly avenue, to enhance performance in an efficient manner.

Those who design devices are urged to work toward agreement among device measurements. Utilizing prediction or correction equations may allow devices to be used interchangeably and ultimately help coaches collect and utilize data in their daily practices more efficiently. 

## Figures and Tables

**Table 1:** Comparison between devices in ground contact time with sprint strike step.

	Optojump Sensor	Pasco force Platform
Mean ± SD	0.104 ± 0.022	0.096 ± 0.023
Pearson correlation	r = 0.979	
Paired *t*-test *p* value	<0.001	
T score (degree of freedom)	16.272 (87)	

**Table 2:** Comparison between devices in ground contact time with drop jump.

	Optojump Sensor	Pasco Force Platform
Mean ± SD	0.251 ± 0.039	0.237 ± 0.038
Pearson correlation	r = 0.993	
Paired *t*-test *p* value	<0.001	
T score (degree of freedom)	27.583 (86)	
